# The Mammalian Metaorganism: A Holistic View on How Microbes of All Kingdoms and Niches Shape Local and Systemic Immunity

**DOI:** 10.3389/fimmu.2021.702378

**Published:** 2021-06-30

**Authors:** Solveig Runge, Stephan Patrick Rosshart

**Affiliations:** ^1^ Department of Medicine II (Gastroenterology, Hepatology, Endocrinology, and Infectious Diseases), Medical Center - University of Freiburg, Faculty of Medicine, University of Freiburg, Freiburg im Breisgau, Germany; ^2^ Faculty of Biology, University of Freiburg, Freiburg im Breisgau, Germany

**Keywords:** bacterial microbiome, mycobiome, virome, archaeome, gut microbiota, lung microbiota, skin microbiota, vaginal microbiota

## Abstract

The field of microbiome research has developed rapidly over the past decades and has become a topic of major interest to basic, preclinical, and clinical research, the pharmaceutical industry as well as the general public. The microbiome is a complex and diverse ecosystem and defined as the collection of all host-associated microorganisms and their genes. It is acquired through vertical transmission and environmental exposure and includes microbes of all kingdoms: bacteria, archaea, prokaryotic and eukaryotic viruses, fungi, protozoa, and the meiofauna. These microorganisms co-evolved with their respective hosts over millions of years, thereby establishing a mutually beneficial, symbiotic relationship on all epithelial barriers. Thus, the microbiome plays a pivotal role in virtually every aspect of mammalian physiology, particularly in the development, homeostasis, and function of the immune system. Consequently, the combination of the host genome and the microbial genome, together referred to as the metagenome, largely drives the mammalian phenotype. So far, the majority of studies have unilaterally focused on the gastrointestinal bacterial microbiota. However, recent work illustrating the impact of viruses, fungi, and protozoa on host immunity urges us towards a holistic view of the mammalian microbiome and the appreciation for its non-bacterial kingdoms. In addition, the importance of microbiota on epithelial barriers other than the gut as well as their systemic effects *via* microbially-derived biologically active compounds is increasingly recognized. Here, we want to provide a brief but comprehensive overview of the most important findings and the current knowledge on how microbes of all kingdoms and microbial niches shape local and systemic immunity in health and disease.

## Introduction

Multicellular organisms are not only composed of their individual cells, but also of the microorganisms that inhabit ecological niches such as the gastrointestinal tract, the skin, the respiratory tract, and the genitourinary system. These microbes do not just passively colonize their hosts, they rather established a symbiotic relationship on all epithelial barrier sites during millions of years of co-evolution. The host provides its resident microbes with a habitat as well as nutrients, and in return, they help in digesting food, providing vitamins, and protecting their host from diseases (1–4).

Microbiota is defined as the ecological community of all host-associated microbes within a particular niche, whereas the collection of those microorganisms and their genomes is known as the microbiome (5). The host genome is the building plan for mammalian organisms, thereby creating well-defined ecological niches for microbes. Nonetheless, the mammalian phenotype itself within a given environment is rather driven by the combination of the host genome and the microbiome together referred to as the metagenome. Therefore, the microbiome plays a pivotal role in virtually every aspect of mammalian physiology, particularly in the development, maturation, homeostasis, and ultimately the function of the immune system (6–11). This mutually beneficial coexistence is also acknowledged in the holobiont theory or the metaorganism concept, emphasizing the intimate relationship between the host and its inhabiting microorganisms (12–14).

Over the last decades, microbiome research has primarily focused on the gastrointestinal bacterial community and its effects on the host health and disease. This line of research provided insights into mechanisms of the bidirectional crosstalk between the host and its bacterial subtenants. However, many disease states are paralleled with changes in the bacterial microbiome and vice versa, leaving the question of whether the microbiome is responsible for the disorder or whether the disease influences microbial composition, placing researchers into a challenging chicken and egg situation. In this context, it is pivotal to acknowledge that the microbiome is a complex and diverse ecosystem comprising microorganisms of all kingdoms, namely bacteria, archaea, eukaryotes like protozoa and fungi, and even multicellular eukaryotes such as helminths (15). In addition, eukaryotic viruses, phages, and endogenous retroviral elements are also crucial members of the mammalian microbiome (16). These microbial communities are present not only in the gut, but at all epithelial barrier sites, and the overall complexity is likely potentiated by trans-kingdom interactions between these commensals (17). As explained above, this complex ecosystem together with the host genome shapes the metaorganism and its physiology through multifactorial and nonlinear interactions. Thus, experimental models of translational research that are aiming to identify causal relationships and to unravel underlying mechanisms must be capable of navigating this complexity. Therefore, focusing on the gut bacteria, merely one component of the entire microbiome, makes it exceedingly difficult to fully decipher complex microbiota-related physiological mechanisms. Instead, a full description of the microbiome at distinct epithelial barrier sites and a more comprehensive view of mammalian organisms as holobionts might be a key to successfully conduct mechanistic studies, thereby opening up a promising window of opportunity to solve the chicken and egg question.

## Technological Challenges in the Analysis of Different Microbial Kingdoms

Ever since, most studies investigating the microbial influence on the host’s physiology focused on bacteria of the gastrointestinal tract, particularly highly abundant bacteria. The primary reason for this bacteriocentric approach was a major technological restriction: pioneer studies on mammalian microbiota almost exclusively relied on culture-based approaches that were more commonly available for bacterial organisms, specifically gastrointestinal microbes. Moreover, the gut incorporates high microbial biomass and is thus much easier to study than niches with low microbial biomass like the skin, the respiratory tract, or the genitourinary system.

Technological advances, especially the common use of sequencing methods and a continuous reduction in the associated costs alongside novel computational bioinformatics tools, have enabled the scientific community to explore the bacterial microbiome as well as other microbial kingdoms in more detail, unveiling previously unappreciated microorganisms (18, 19). However, the focus on bacterial organisms of the gut remained vastly unchanged. Thus, the commonly used methods were optimized for detecting bacterial DNA, which causes problems when focusing on viruses, archaea, or eukaryotes like fungi. When using 16S rRNA gene profiling, microorganisms other than bacteria are vastly neglected as the primers fail to cover multi-kingdom diversity. Nowadays, the probably least biased approach to study highly diverse microbial communities is shotgun metagenomic sequencing. However, the predominance of bacteria leads to an unfavorable bacteria-to-archaea or bacteria-to-fungi ratio, and thus a high sequencing depth is needed to detect comparably rare fungal or archaeal signatures (20, 21). The differences in the cellular structures between bacteria, archaea, or eukaryotes are another technological hurdle in analyzing microbial DNA. For example, commercial DNA extraction kits contain lysozyme that cuts bacterial peptidoglycan but not archaeal pseudopeptidoglycan (21). Likewise, the efficiency of isolating fungal DNA significantly differs between several isolation methods for microbial DNA (22). Another challenge comes when analyzing viral communities, as the genome of many viruses is not composed of DNA but RNA, so that the study design requires the inclusion of RNA in its analyses (23). Thus, the research community urgently needs tailored protocols to enrich, extract and study non-bacterial kingdoms such as the archaeome, the virome, the mycobiome, and other eukaryotes like protozoa and the meiofauna.

Another crucial component to ensure future success in microbiome research will be the availability of high-quality databases. Although current databases for bacteria are already refined and accurate, they certainly need further optimization. This is substantially different for other microbial kingdoms, as reliable and well-annotated databases are urgently needed for analyses at the species level, but often not available (21, 24). Many sequences included in databases for fungal communities are annotated as uncultured or are incorrect at the species level (25). This is further complicated by the fact that sexual and asexual forms of the very same fungal species are frequently classified as different taxa (26, 27). In virome studies, only around half of the sequences can be aligned to reference databases, indicating an enormous amount of viral “dark matter” that needs further exploration (23, 28). Hence, the scientific community imperatively needs further optimization of databases, particularly as regards non-bacterial kingdoms. This could be achieved by studies shedding more light on the “dark matter” still obscuring vast parts of the mammalian microbiome, thereby empowering mechanistic studies on host-microbe interactions.

## The Microbiome of the Gastrointestinal Tract: Local Effects on Immunity

### Gut Bacterial Microbiome

**Figure 1:**
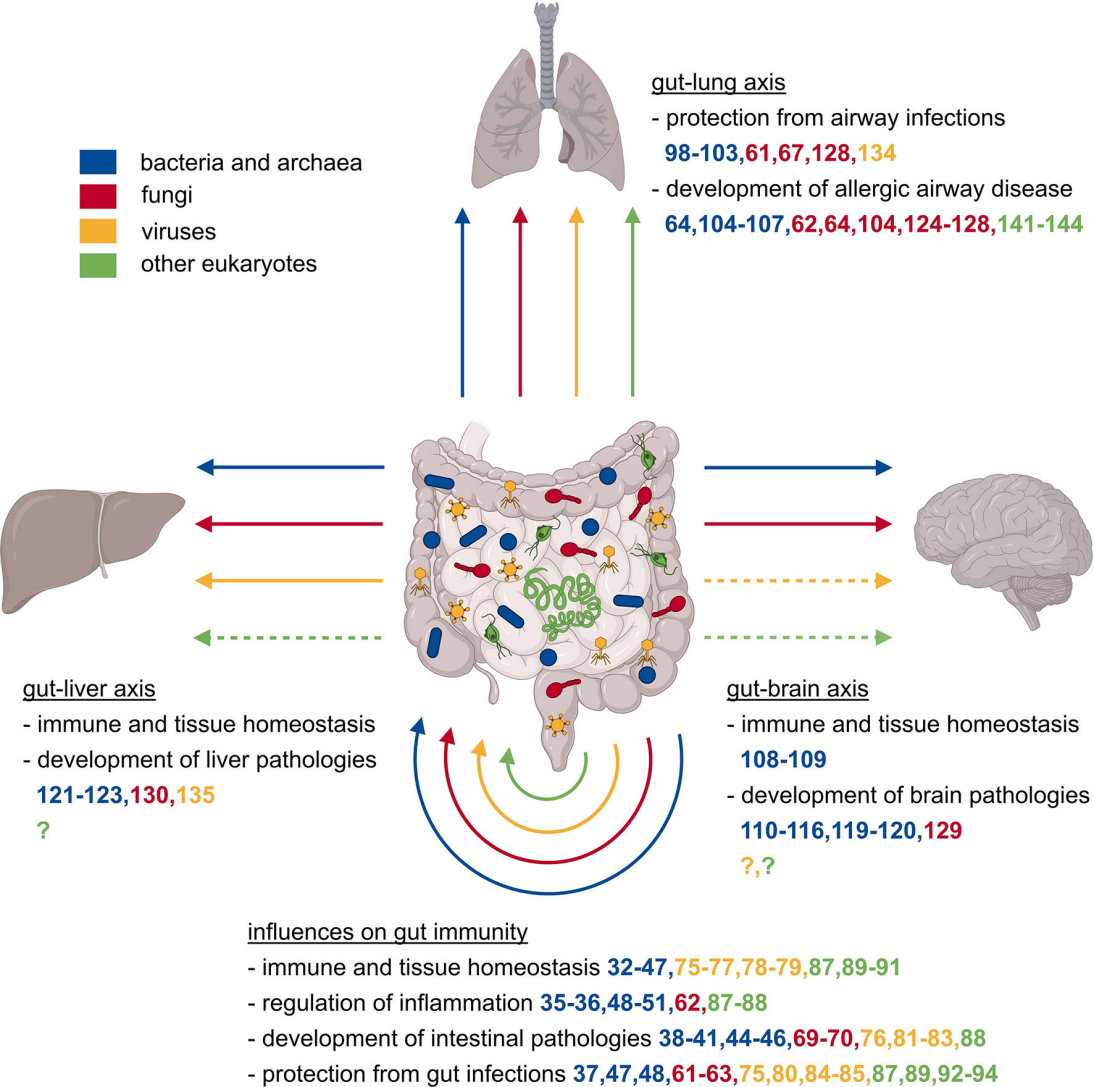
Local and systemic effects of the gut microbiome on the immune system. The mammalian gut is populated by a plethora of microbes comprising representatives of all kingdoms, specifically bacteria, archaea, eukaryotes like fungi, protozoa, and helminths, as well as eukaryotic viruses and bacterial phages. The collectivity of all gut-associated microorganisms affects the development, maturation, homeostasis, and consequently functionality of the immune system, which in turn has local and systemic consequences. Changes in the microbial composition might affect the protection against or acceleration of inflammatory diseases, regulation of intestinal pathologies, and the protection against intestinal infections. Microbiota influence not only the gut tissue itself, but also other organs like the liver, lung, and brain *via* the so-called gut-liver, gut-lung, or gut-brain axes. These distant organs are also affected positively or negatively in their homeostasis, defense against infections, or development of organ-specific pathologies.

The gastrointestinal tract certainly is the most intensively studied microbial niche and considering its impact on local and systemic immunity, the most remarkable epithelial barrier site of mammalian organisms. Further, the bacterial microbiome of the gastrointestinal tract is the so far best characterized microbial community that mainly comprises anaerobic bacteria of the phyla Bacteroidetes and Firmicutes (29). It plays an essential role in the development, maturation, aging, and homeostasis of the host’s immune system and thus the orchestration and functionality of host immune responses in steady state and during various inflammatory events (**Figure 1**) (30, 31).

Several studies showed that certain gut bacteria strongly shape local immunity at the corresponding barrier site. An example are segmented filamentous bacteria (SFB), gram-positive bacteria that grow close to the intestinal epithelium and that induced the formation of T helper 17 (T_H_17) cells, which are crucial for tissue homeostasis at barrier sites (32). However, not only commensal SFB but also other bacteria like murine enteropathogenic *Citrobacter rodentium*, a human pathogenic *Escherichia coli* strain, or the human symbiont *Bifidobacterium adolescentis*, were able to induce an accumulation of T_H_17 cells (33, 34). Adhesion to the intestinal epithelial cells seems to be a key factor for gut T_H_17 cell differentiation, as this is a shared feature of the above-mentioned bacteria, and adhesive-defect mutants did not induce T_H_17 cell development (33, 34). However, further studies suggested that T_H_17 cells induced by different bacteria display divergent inflammatory phenotypes (35). SFB are not only important in T_H_17 responses but also seem to induce T follicular helper cell development in the Peyer’s patches, which leads to the aggravation of inflammation in an autoimmune arthritis model (36). Not only bacteria themselves, but also their products such as metabolites affect immune development and functionality. For instance, microbiota-derived short-chain fatty acids (SCFA) are needed for antigen-activated CD8^+^ T cells to develop into long-living memory cells (37). SCFA produced by *Clostridia* of the Cluster IV, XIVa, and XVIII promote the accumulation of interleukin (IL)-10 producing regulatory T (T_reg_) cells in the colon and attenuate pathology in a colitis model (38–40). A bacterial polysaccharide of *Bacteroides fragilis* also induced IL-10 producing T_reg_ cells, which protect against experimental colitis induced by *Helicobacter hepaticus* (41). Bile acids, further microbial-derived metabolites, play a protective role in the intestine by interacting with the farnesoid X receptor (FXR) (42, 43). Additionally, recent studies suggested bacterially produced secondary bile acids to regulate the differentiation of colonic T_reg_ cells, which have a positive impact on dextran sulfate sodium (DSS)-induced colitis (44–46). Not only the gastrointestinal T cell compartment but also B cell development in the intestinal mucosa is affected by commensal colonization (47). Other microbial metabolites, like aryl hydrocarbon receptor (AhR) ligands, produced by tryptophan-metabolizing microbes, induce the production of IL-22, thereby providing resistance to *Candida* colonization and gut inflammation (48). Additionally, various microbial molecules can influence macrophage polarization towards either a pro-inflammatory or an anti-inflammatory state, depending on the stimulating metabolites and the context (49–51). Gut-resident bacteria and their metabolites thus have a substantial effect on gastrointestinal health and disease.

### Gut Archaeome

Over 50 years ago, the first methanogenic archaeal microorganism was isolated from human feces, ever since we know that the gastrointestinal tract is also inhabited by representatives of the archaeal domain (52). Further methanogenic and to a lesser extent also non-methanogenic archaeal species were detected in the human gut, supporting initial findings that methanogenic archaea are commensal inhabitants of the human gastrointestinal tract (53). Some methanoarchaeal species showed immunogenic properties (54, 55), however, the interaction between archaea and the immune system, as well as the involvement of archaea in human physiology remains blurry (21). A recent study suggested trans-kingdom interactions between bacteria of the *Christensenellaceae* family and archaea of the *Methanobrevibacter* family to be associated with a lean body mass index in humans (56). Interestingly, no human pathogenic archaeal species are known today, and there is only little to no data available on how archaea might affect the physiology of their respective hosts (21). Consequently, a substantial amount of research is required to further unravel the relationship between the host and its archaeal inhabitants.

### Gut Mycobiome

The gut mycobiome is a diverse community mainly comprising *Saccharomyces*, especially *Candida* species, as well as yeasts of the family *Dipodcaceae*, and occasionally also *Malassezia* and *Cladosporium* (57–59). Comparable to the gut bacterial microbiome, gastrointestinal fungi were also shown to strongly shape local immunity at the corresponding barrier site (60). *Candida albicans* colonization in the gut induced IL-17 and IL-22 production of T_H_17 cells that protect from systemic infection with *Candida albicans* and *Staphylococcus aureus* (61, 62). However, *Candida albicans* gut colonization also promoted susceptibility to T_H_17 cell-mediated airway inflammation and correlated with systemic levels of T_H_17 cell inflammation (62). In humans, the gut mycobiome induced antifungal antibody production that protects against systemic fungal infections in a caspase recruitment domain-containing protein 9 (CARD9) dependent way (63). Commensal fungi seem to have certain anti-inflammatory properties as a disturbance of the fungal gut community aggravated colitis in a mouse model (64). Similarly, the interaction of gut fungi with C-type lectin receptors on macrophages seems to protect from DSS-induced colitis and colon cancer (65, 66). Fungal colonization after antibiotic treatment can even recapitulate the beneficial effect of bacterial gut colonization on DSS-induced colitis (67). However, these anti-inflammatory capabilities appear to be context-sensitive, since fungal colonization seemed to be detrimental in colitis in specific-pathogen-free (SPF) mice (68). Patients suffering from Crohn’s disease frequently show elevated anti-*Saccharomyces cerevisiae* antibodies (ASCA), leading to the assumption that commensal fungi might play a role in human gastrointestinal diseases (69). Importantly, ASCA can even be detected before disease onset and are highly predictive for receiving a diagnosis of Crohn’s disease within the next five years (70). Thus, the gut mycobiome also appears to be an essential component in shaping local immunity in steady state as well as during inflammation.

### Gut Virome

The gastrointestinal virome also plays a pivotal role in shaping local gut immunity. A substantial proportion of viruses detected in fecal samples are bacteriophages that are likely to influence the composition and functional properties of the gastrointestinal bacterial community (71). Phages might do so by predating susceptible bacterial strains, which confers an advantage to the growth of others (72). Another mechanism is horizontal gene transfer that changes genetic diversity and thereby influences virulence, antibiotic resistance, and metabolic determinants of the bacterial community (73). Thus, phages capable of affecting the composition of gastrointestinal bacterial communities exert an indirect impact on the function of the immune system (72, 74). This is further illustrated by a study that suggested bacteriophage diversity and colonization level to be a key factor in the success of fecal microbiota transplantation (FMT) in the therapy of recurrent *Clostridium difficile* infection (75). Besides their indirect effects, phages can also directly exacerbate colitis by inducing host immunity *via* toll-like receptor (TLR)-9 stimulated interferon (IFN)-γ production (76). Bacteria-infecting prophages initiate viral gut colonization during the first months of life; eukaryotic viruses follow a few months later, depending on breastfeeding (77). The presence of enteric viruses in the gut is imperative for tissue homeostasis and the prevention of overt inflammation. Type I IFN stimulated by activation of the viral pattern recognition receptors (PRRs) retinoic acid-inducible gene I (RIG-I) and stimulator of interferon genes (STING) is protective against intestinal barrier damage and prevented graft-versus-host disease (GvHD) in mice (78). RIG-I activated by enteric viruses also stimulates IL-15 secretion, which is important for maintaining tissue-regenerative intraepithelial lymphocytes (79). Another virus-sensing PRR, TLR7, enhances resistance to vancomycin-resistant *Enterococcus* infection of antibiotic-treated mice *via* secretion of anti-microbial peptides stimulated by IL-22 producing innate lymphoid cells (ILC) (80). Recognition of gut viruses by TLR3 and TLR7 also protects against DSS-induced colitis *via* a type-I IFN mediated mechanism (81). IFN-λ induced by enteric viruses was suggested as an additional protective factor against DSS-induced colitis by preventing reactive oxygen species (ROS) production and neutrophil degranulation (82). Infection with *murine norovirus* could even compensate for the absence of gut bacteria regarding their immune-promoting function and the protective capacities against *Citrobacter rodentium* induced pathologies (83). Another study found *murine astrovirus*, a commensal gastrointestinal virus in mice, to protect against *murine norovirus* and *rotavirus* in immunodeficient mice by increasing IFN-λ levels (84). This effect is mediated by stimulation of type I IFN that leads to the recruitment of C-C chemokine receptor type 2 (CCR2) dependent monocytes and the production of IL-22 by type 3 ILC (85). Hence, besides gut bacteria and fungi, the gut virome is also a crucial factor for immunity at the barrier site, not only by direct interaction but also indirectly by affecting other microbial kingdoms. This is a relevant and increasingly recognized phenomenon known as trans-kingdom interactions (17).

### Other Eukaryotic Members of the Gut Microbiome

The impact of eukaryotic multicellular organisms like protozoa and helminths on local and systemic immunity is still largely unrecognized (86). Some organisms such as *Entamoeba histolytica* and *Ascaris lumbricoides* are obligatory pathogens, while others, for example, *Blastocystis*, are associated with disease but also found in healthy people (86). The commensal murine protist *Tritrichomonas musculis* induced an accumulation of T helper 1 (T_H_1) and T_H_17 cells in an inflammasome and IL-18 dependent mechanism (87). This induction of adaptive immunity enhanced anti-bacterial defense but also increased intestinal inflammation (87). Similarly, *Tritrichomonas muris* was found to induce a T_H_1 response in the cecum leading to accelerated gastrointestinal inflammation in a colitis mouse model (88). Different studies found the SCFA succinate, produced by *Tritrichomonas muris*, to activate tuft cells in the intestinal epithelium. Tuft cells subsequently induced the secretion of IL-13, IL-4, and IL-5 by type 2 ILC in an IL-25 dependent manner (89–91). This cytokine milieu promoted the expansion of tuft and goblet cells, a common mechanism for clearing helminth infection and protecting from subsequent colonization by other parasites (89). Interestingly, tuft cell expansion promoted *norovirus* infection as the *norovirus* receptor is specifically expressed on tuft cells (92). Moreover, helminth infection can manipulate the immune system leading to an impaired antiviral immunity or even viral reactivation (93, 94). Non-fungal eukaryotes are regularly found in the gut and seem to have beneficial as well as detrimental effects on local immunity by deteriorating intestinal inflammation but also preventing secondary helminth infection.

Taken together, a rapidly growing body of literature points towards a pivotal role of the microbial community including representatives of all microbial kingdoms in the homeostasis of the gut. Particularly, the development, maturation, aging, and thus the functionality of host immune responses at the barrier site in steady state as well as during various inflammatory events are largely orchestrated by the gut microbiome.

## The Microbiome of the Gastrointestinal Tract: Systemic Effects on Immunity

### Gut Bacterial Microbiome

The gastrointestinal bacterial microbiome does not only shape immunity at its corresponding barrier site but also exerts powerful effects on systemic immune responses (**Figure 1**). Among the most affected organs are the lung, the brain, and the liver whose physiology is influenced *via* the so-called gut-lung (95), gut-brain (96), or gut-liver axis (97). The gut-lung axis has a significant influence on the susceptibility to respiratory infections and allergic airway diseases. For example, germ-free mice are more susceptible to lethal *Klebsiella pneumoniae* infection, an effect that can be reversed by transient TLR activation through administration of several TLR agonists usually produced by indigenous microbiota (98). A comparable effect of a reduced microbial load was observed in the context of *Influenza A virus* (IAV) infection. Mice pre-treated with antibiotics showed an aggravation of IAV infection due to a diminished T cell response because of poorly activated dendritic cells (DC) and a higher activation threshold of innate immunity (99, 100). Additionally, TLR5 activation by bacterial flagellin in the gut is essential for the development of an efficient antibody response to IAV vaccination (101). Another study found the metabolite desaminotyrosine produced by *Clostridium orbiscindens* to be a protective factor against IAV infection by modulating type I IFN signaling (102). A further mechanism that might prevent overt immunopathology in the lung following IAV infection was SCFA-mediated alteration of bone marrow hematopoiesis leading to increased numbers of anti-inflammatory macrophages in the lungs (103). Gastrointestinal bacteria not only play a pivotal role during respiratory infections, but they also appear to be crucial in allergic airway disorders. Gut dysbiosis induced by antibiotic or antifungal treatment can aggravate allergen-induced airway inflammation (64, 104). Otherwise, infection with *Helicobacter pylori* conferred protection towards experimentally induced lung inflammation, a T_reg_ cell-mediated effect (105, 106). A comparable protection from allergic airway disease was found in mice supplemented with *Lactobacillus johnsonii*, a bacterium that is enriched in the gastrointestinal tract of mice that were previously exposed to house dust mites (106). However, the protective effect of the bacterial microbiome might be reversed in the context of chronic pulmonary diseases. A study using a mouse model of cystic fibrosis showed amelioration of airway hyperresponsiveness after lowering the enteric bacterial burden by antibiotic treatment with Streptomycin (107).

Besides the lung, the gut bacterial microbiome is very well known to affect the development, maturation, normal aging, homeostasis, and function of the brain, which is accomplished through communication along the gut-brain axis (96). Important mediators for this are neuroactive metabolites produced by gut-resident microbiota. Good examples are the influence of gastrointestinal bacteria on microglial development and homeostasis, an important phenomenon likely mediated through microbially-produced, neuro-modulatory SCFA (108). This study also highlights that the complex and diverse gut community, rather than single gut-resident bacteria, is fundamental for proper microglial development and function (108). Not only microglial development but also axogenesis is affected by gut bacteria and their metabolites. Axogenesis was markedly reduced in offspring of germ-free or antibiotics-treated damns, leading to sensation impairment in the offspring (109). This effect could be reversed by colonizing the pregnant damns with spore-forming bacteria or treatment with selected metabolites (109). The microbiota-mediated influence on brain homeostasis also affects the progression of several brain pathologies including psychiatric disorders like autism spectrum disorder (ASD), as well as neurological diseases such as Alzheimer’s disease (AD), Parkinson’s diseases (PD), multiple sclerosis (MS), and stroke. To mention some examples, microbiota depletion attenuated brain inflammation and pathologies in mice with experimental autoimmune encephalitis (EAE), a gold-standard model of human MS as well as in an AD mouse model (110–112). Susceptibility to developing EAE symptoms could be transferred from MS patients to transgenic mice spontaneously developing EAE, which showed an increased incidence of disease symptoms after FMT from MS diseased donors (113). Moreover, in a mouse model of ASD, oral treatment with *Bacteroides fragilis* ameliorated ASD-related behavioral abnormalities, an effect that is most likely mediated by microbiota-dependent metabolites (114). Another study using a PD mouse model has underlined the microbial influence on neurological disorders by identifying gut microbiota to enhance α-synuclein-mediated motor dysfunction, an effect probably also mediated by microbially-produced SCFA (115). Further, the gut bacterial microbiota affects meningeal IL-17 producing gamma delta T (γδT) cells, which worsened the outcome of experimentally induced strokes (116). Similar to the intestine, another part of the digestive tract, the oral cavity, has a niche-specific bacterial microbiome (117). Interestingly, the amount and diversity of oral colonization seem to decline during weaning, coinciding with upregulation of saliva production and salivary antimicrobial components (118). Moreover, in several studies, periodontitis and tooth loss were associated with the development of dementia and Alzheimer’s disease (119, 120).

There also is an interplay between the gut bacteria and the liver. So, common liver diseases like alcoholic liver disease (ALD), non-alcoholic fatty liver disease (NAFLD), liver cirrhosis, or hepatocellular carcinoma (HCC) are associated with changes in the bacterial gut microbiome (97). Changes in the microbiome can also change liver cancer by bile acids transformation that affect CXCR6^+^ natural killer T (NKT) cells in the liver (121). A therapeutic option using the microbiome-related effect on liver immunity is FMT, which showed promising results in an ALD mouse model and was recently also explored in a first clinical trial in humans (122, 123). Even though, science has already gained substantial knowledge on how gut bacterial microbiota influence immunity in distant organs, there is still extensive research required to fully decipher these complex interactions.

### Gut Mycobiome

Like the gut bacterial microbiome, the gut mycobiome can also shape systemic immune responses, particularly in the lung, brain, and liver. Regarding the gut-lung axis, fungal dysbiosis caused by antibiotics or antifungal treatment can exacerbate experimentally induced allergic airway disease (64, 124). Allergic airway inflammation was significantly aggravated through sensing of fungal dysbiosis by gut resident mononuclear phagocytes (MNPs) and a subsequent increase in pulmonary T helper 2 (T_H_2) cells and eosinophils (125). The beneficial effect of fungi on allergic airway disease seems to be a tightly balanced equilibrium, as fungal overgrowth following antibiotic treatment leads to a promotion of allergic airway inflammation (126). One of the proposed mechanisms was the elevation of prostaglandin E2 (PGE2) plasma levels by the overgrowth of *Candida* species and the promotion of macrophage polarization towards an alternatively activated M2 phenotype (104). Additionally, the expansion of a single fungal species in the gut, *Wallemia mellicola*, could aggravate allergic airway disease (127). In humans, *Candida albicans*, a common gut fungal species, seems to be the major direct inducer of anti-fungal T_H_17 cells in peripheral blood (61). These T_H_17 cells are cross-reactive to inhaled *Aspergillus fumigatus* and are activated and expanded in patients with airway inflammation (61). Likewise, intestinal colonization with *Candida albicans* in mice promoted susceptibility to airway inflammation (62). *Candida albicans* strains adapted to the mouse gastrointestinal tract conferred enhanced protection against systemic infection with several fungi and bacteria, but this effect required IL-6 and was also observed in lymphocyte-deficient mice (128). Moreover, fungal colonization following antibiotic treatment recapitulates the beneficial effect of bacterial gut colonization in lethal IAV infection (67).

Like gut bacteria, fungi can also affect brain and liver homeostasis. An example of this is the improvement of symptoms of EAE, after oral supplementation of mice with *Candida kefyr* (129). Regarding influences on the liver, gut-resident fungi promote the development of ALD, an effect that is mediated by increased translocation of fungal β-glucan to the systemic circulation, which induced IL-1β-mediated liver inflammation through binding to C-type lectin domain family 7 member A (CLEC7A) on Kupffer cells (130).

Furthermore, the gut mycobiome has a fundamental influence on the maturation of the immune system itself. The gastrointestinal fungal species *Candida tropicalis* was found to play a substantial role in the early life maturation of secondary lymphoid organs (131). Recent studies transferring mice into a natural environment supported this by showing an increased fungal diversity and an elevated fungal load, especially in *Aspergillus* species. These alterations of the fungal community were accompanied by an increase in peripheral granulocytes and activated T cells, illustrating an enhanced immune maturation (132, 133). Not only gut bacteria, but also gut fungi have systemic effects on immunity. Even though there is only limited data on fungal metabolites, their involvement in this communication seems likely.

### Gut Virome

The gastrointestinal virome also shapes immunity at gut-distal sites such as the lung and the liver. For example, an infection with *murine norovirus* protects against lung infection with *Pseudomonas aeruginosa* and alleviates lung inflammation (134). A liver-related example is that a decreased diversity of the intestinal virome is associated with an increase in the severity of NAFLD in humans (135). Further research is required to fully understand how intestinal viral communities shape peripheral immune reactions at various sites.

In addition to gut-resident viruses, there is increasing evidence that systemic chronic viral infections are not only pathogenic but can also have advantages to the host (136). Latent herpesvirus infection mediates resistance to bacterial infection with *Listeria monocytogenes* or *Yersinia pestis* and results in increased resistance to tumor grafts (137–139). Type I IFN production induced by chronic murine cytomegalovirus (MCMV) infection stimulated epithelial proliferation and intestinal wound repair (140). Latent infection with chronic viruses has thus been considered as an integral part of the microbiome that has a substantial influence on the host’s immune system.

### Other Eukaryotic Members of the Gut Microbiome

Apart from local effects on the gastrointestinal immune system, multicellular eukaryotes were also found to influence immunity in the lung. In mouse models, infection with *Heligmosomoides polygyrus* leads to a decreased development of allergic airway inflammation, probably through a mechanism that involves T_reg_ cells and is independent of IL-10 (141). Interestingly, *Heligmosomoides polygyrus* derived egg-shell products were sufficient to prevent experimental allergic airway inflammation, probably by directly inhibiting IL-33 release (142, 143). In humans, reports are still inconsistent regarding a correlation between gastrointestinal helminth infection and the occurrence of allergic airway disease (144). More research on protist and helminth members of the gut microbiome is required for a better description of their influence on immunity.

Taken together, the gut bacterial microbiome exerts many important effects on various gut-distant organs such as the lung, the brain, and the liver. However, current data also clearly indicate a critical involvement of nearly all microbial kingdoms in the functionality of the host’s immune system, and thus the orchestration of systemic host immune responses in health and disease. Importantly, biologically active microbial compounds appear to be essential in mediating the communication between the host and its microbiota along the corresponding axis.

## The Microbiome of the Skin: Local and Systemic Effects on Immunity

### Skin Bacterial Microbiome

**Figure 2:**
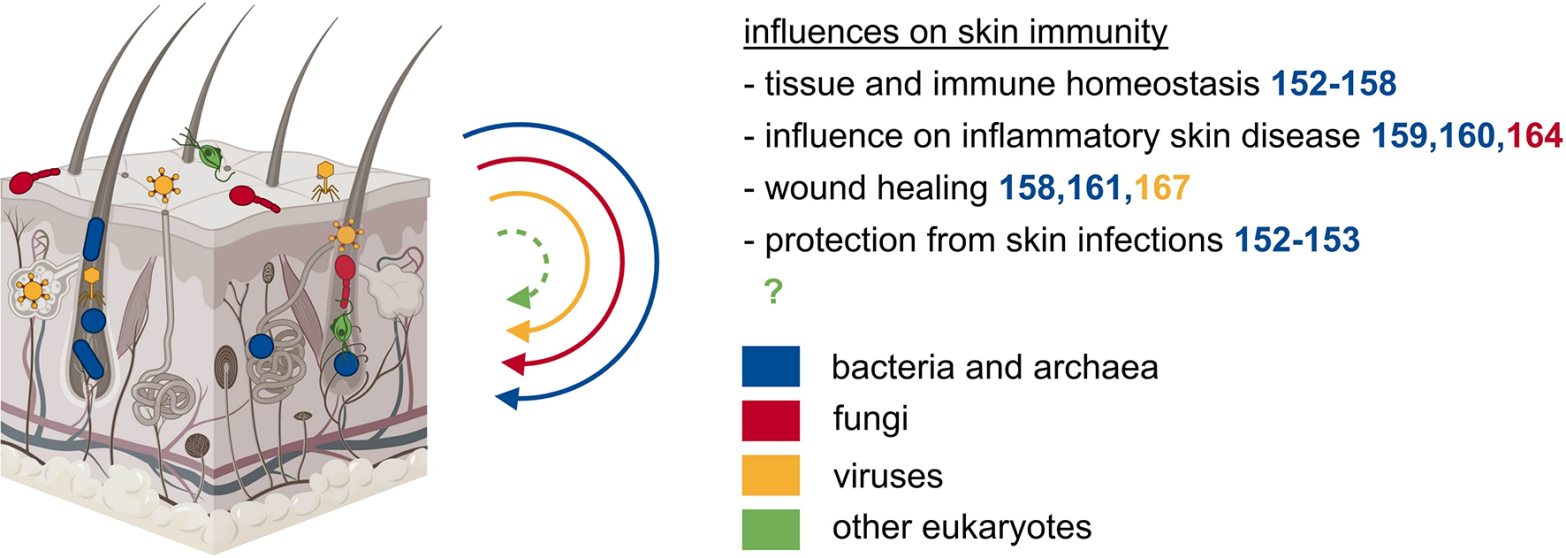
The microbiota of the skin exerts local effects on immunity. Besides the gut, also other epithelial barrier sites such as the skin are populated by diverse microbes of all kingdoms. The microbiota of the skin influences tissue and immune homeostasis, inflammatory skin diseases, wound healing, and protects from skin infections.

The skin is the outermost barrier of our body and is in constant contact with multiple environmental influences. To maintain this barrier, our skin works together with its residing microbiota (**Figure 2**) (145, 146). Various species are adapted to the specific properties of the respective site and thus inhabit different cutaneous microenvironments (147). For example, sebaceous sites are dominated by lipophilic *Propionibacterium* species, while *Staphylococcus* and *Corynebacterium* species colonize the moist areas (148). The initial microbial skin colonization depends on the delivery mode. Vaginally delivered babies acquire the mother’s vaginal microbiome, while cesarean section leads to the acquisition of skin-associated microbiota (149, 150). A major shift in microbial skin communities occurs during puberty: several taxa disappear and the microbiota becomes dominated by lipophilic species (151). During cutaneous immune homeostasis, skin commensal bacteria maintain the host-microbial mutualism by protective and regulatory responses. Commensal bacteria affect the immune system in the absence of inflammation and independent of changes in the gut microbiome (152, 153). Cutaneous colonization with the skin commensal *Staphylococcus epidermidis* leads to a non-inflammatory accumulation of IL-17A and IFN-γ expressing CD8^+^ T cells in the skin (152). Similarly, T cells in germ-free mice produced significantly lower cytokine levels, a phenotype that could be reversed by colonization with *Staphylococcus epidermidis* (153). Tolerance to commensal microbes is established during the postnatal period when developing hair follicles are colonized by microbes that induce commensal-specific T_reg_ cells (154, 155). The complement system, which is part of this regulatory mechanism by maintaining microbial diversity, is also regulated by commensal microbes (156). The commensal *Staphylococcus epidermidis* seems to be an essential microbe capable of regulating immunity at this barrier site. In an *in vitro* study using human monocyte-derived DC, *Staphylococcus epidermidis* products stimulate DC to produce more IL-10 and lower the proliferation effect on CD4^+^ T cells (157). Additionally, T_reg_ cells treated with Staphylococcus products have a higher immune-suppressive potential on T cells (157). Interestingly, this homeostatic immunity to microbiota is mediated by non-classical MHC class I molecules (158). Metabolites of commensal bacteria also have a suppressive effect on the development of skin inflammation. Treatment of atopic dermatitis with lysates of *Vitreoscilla filiformis*, a gram-negative bacterium present in thermal spa water, leads to pronounced amelioration of atopic dermatitis symptoms in a clinical trial and an experimental model (159, 160). Studies in mice suggested that this is an effect of IL-10 secretion from DC and accumulation of T_reg_ cells, which have a suppressive effect on T cell expansion (160). The bacterial skin microbiome also affects the process of acute wound healing, specifically when the integrity of the skin barrier is breached. During inflammation caused by skin wounding, lipoteichoic acid of *Staphylococcus epidermidis* can mitigate skin inflammation in a TLR dependent manner (161). Wound closure is also accelerated by commensal-specific T cells that express tissue repair and immunoregulatory signatures (158). The influence of commensal microbes on local immunity confers resistance to potentially harmful pathogens. *In vivo*, skin colonization with *Staphylococcus epidermidis* led to CD103^+^ DC dependent formation of IL-17 producing CD8^+^ T cells leading to the production of alarmins by keratinocytes inhibiting *Candida albicans* outgrowth (152). Restoration of IL-17 production after *Staphylococcus epidermidis* colonization also helped in the control of *Leishmania major* infection but then again lead to an increased inflammatory response (153). In summary, the skin bacterial microbiome is a fundamental player in securing skin homeostasis, preventing inflammatory skin diseases, supporting wound healing, and protecting against pathogenic infections.

### Skin Archaeome

Besides multiple bacterial species, archaea of the phyla Thaumarchaeota and Euryarchaeota were detected on human skin samples (53, 162). Their abundance is correlated to dry skin occurrence, but mechanistic details of a potential causal relationship are missing (163).

### Skin Mycobiome

Lipophilic fungi of the genus *Malassezia* are the dominant fungal microorganisms on the skin of most adults (148, 151). A recent publication suggested that immunity to commensal fungi might play a role in the aggravation of skin inflammation in psoriasis (164). Thus, recent literature shows an important role of fungi in skin immunity, thereby prompting us to more closely investigate the role of the skin mycobiome.

### Skin Virome

Eukaryotic viruses and phages are detected on human skin and are the most unstable part of the skin microbiome (165, 166). Bacteriophages were found to negatively interfere with chronic wound healing in *Pseudomonas aeruginosa* infected wounds, likely mediated by endocytosis and TLR3 (167).

In conclusion, even though microorganisms other than bacteria are highly abundant on the skin and their involvement in skin immunity has been shown in some promising studies, their local and especially systemic influence on the host’s immune system is still poorly explored. However, unraveling these interactions appears to be a promising field for future studies.

## The Microbiome of the Respiratory Tract: Local and Systemic Effects on Immunity

### Respiratory Tract Bacterial Microbiome

**Figure 3:**
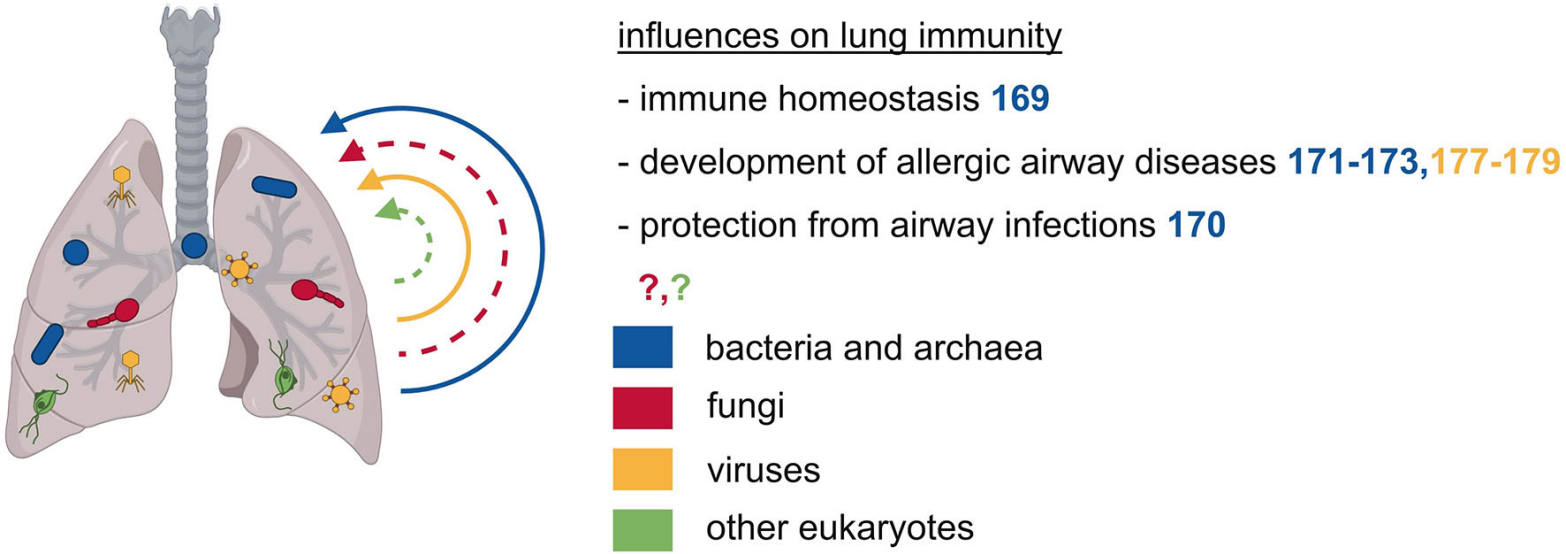
The microbiota of the respiratory tract exerts local effects on immunity. The respiratory tract is also inhabited by various microbes of different kingdoms. These microbes can prevent allergic airway diseases such as asthma and they can protect from airway infections.

The initial dogma of the lung being devoid of microorganisms has recently shifted by advances in sequencing techniques, leading to improved microbial detection. These findings also indicate that lung immunity is influenced by the lung microbiome in several diseases (**Figure 3**). The respiratory bacterial microbiome differs markedly between the upper and lower respiratory tract and is probably influenced by the oral microbiome through frequent micro-aspirations and by airborne microorganisms present in the inhaled air (168). Enrichment of the bacterial lung microbiota with oral taxa is associated with an increased number of lymphocytes, especially T_H_17 cells, elevated cytokine levels, and a diminished TLR4 response by alveolar macrophages, thus influencing the basal inflammatory status in the lung (169). *Staphylococcus aureus* in the lung leads to TLR2-dependent recruitment of monocytes that differentiate into alveolar macrophages and exert a protective effect against IAV pathologies (170). The presence of lung microbiota in early life is essential for the immune maturation and attenuation of airway inflammation that is mediated by the interaction of T_reg_ cells with programmed cell death 1 ligand 1 (PD-L1) expressing DC (171). A similar effect could be observed in children growing up on farms in Central Europe, that were protected from asthma and atopy supposedly due to an increased environmental microbial exposure (172). In line with this, maternal exposure to *Acitenobacter lwoffii* F78, a bacterium frequently found in cowsheds and farm dust samples leads to the protection of the offspring against experimental asthma (173). Thus, lung bacterial communities may mediate a regulatory function in the development of allergic airway diseases.

### Respiratory Tract Archaeome

The lung is also colonized by archaeal microbes, mainly by *woesearchaeal* species (53). However, there is little to nothing known about the function and effects of archaea in the respiratory tract.

### Respiratory Tract Mycobiome

There is only a low fungal burden in the lungs, consisting primarily of the division of *Ascomycota* and *Basidiomycota* with *Candida species* being the predominant organisms (174). So far, there is only sparse knowledge about fungal influences on the host’s immunity in the respiratory tract.

### Respiratory Tract Virome

The virome in the respiratory tract is composed of intrinsic components with a high abundance of *Anelloviruses* and phages, and other respiratory viruses that are normally considered pathogens (175, 176). Early life infection with IAV protects against airway hyperreactivity, a protective effect primarily mediated through an expansion of NKT cells (177). On the contrary, early infection with respiratory syncytial virus increases susceptibility to allergic airway disease by impairing T_reg_ cell function (178). Another study in mice found that infection with Sendai virus is associated with subsequent airway hyperreactivity that depends on IL-13 dependent activation of NKT cells and lung macrophages (179). Overall, there is only little knowledge regarding the impact of the lung-resident virome on local or systemic immunity.

In summary, the currently available data on lung microbiota are still limited, particularly as regards to microorganisms other than bacteria. This is likely due to the low microbial biomass present in the lung, thereby leading to profound technical challenges in reliably assessing the lung microbiota. However, studying the lung microbiome certainly is an interesting future topic, since the lung is the second largest barrier site and is affected by various diseases where the microbiome is known to be a crucial factor (e.g. infectious diseases, cancer, allergies, inflammatory and autoimmune diseases).

## The Microbiome of the Genitourinary Tract: Local and Systemic Effects on Immunity

### Genitourinary Tract Bacterial Microbiome

**Figure 4:**
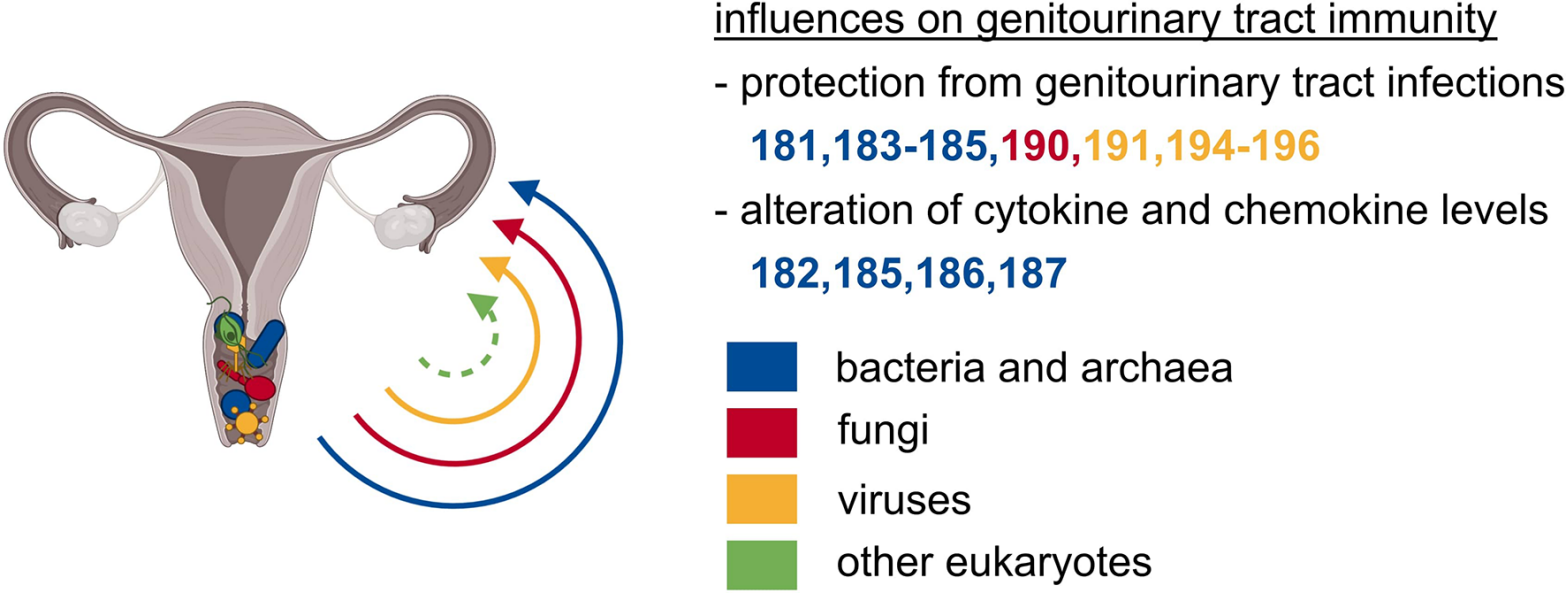
The microbiota of the genitourinary tract exerts local effects on immunity. Particularly the vagina is colonized with abundant microbes of unrelated kingdoms. The vaginal microbial communities safeguard from local, ascending, and subsequently systemic infections, and are crucial for beneficially balanced cytokine as well as chemokine profile.

Similar to other microbial niches, the genitourinary tract is also colonized by microorganisms of different kingdoms that have direct or indirect effects on immunity (**Figure 4**). Compared to other microbial niches, the vaginal bacterial microbiome is a relatively basic community, characterized by comparably low diversity and is dominated by *Lactobacillus* species, which thrive in this anaerobic environment (24, 180). Some asymptomatic women carry a more diverse vaginal community containing bacteria of the genera *Gardnerella* and *Prevotella* (24, 180). However, *Lactobacilli* play the most pivotal role in maintaining the homeostasis of the vaginal tract through the production and secretion of anti-microbial compounds such as H_2_O_2_, bacteriocin, and lactic acid. These compounds serve as a first, effective line of defense by establishing a low vaginal pH and by creating an overall hostile environment for invading bacteria and other microorganisms (181). Another direct effect of lactic acid is its anti-inflammatory impact on vaginal epithelial cells, shown by a reduced production of pro-inflammatory cytokines and increased production of the IL-1 receptor antagonist (IL-1RA) (182). Therefore, a *Lactobacilli*-dominated vaginal microbiome protects against urogenital infections such as urinary tract infections and sexually transmitted pathogens like *Chlamydia*, *human immunodeficiency virus* (HIV), and *herpes simplex virus 2* (HSV-2) (183–185). The increased occurrence of HIV infection in women with a high-diversity vaginal bacterial microbiome might be explained by an increased number of activated CD4^+^ T cells, also including CCR5^+^ CD4^+^ T cells, the HIV target cells (185). The increase in activated T cells might be induced by elevated cytokine and chemokine levels in the genitourinary tract of women with high-diverse vaginal bacterial communities (185, 186). The increased abundance of pro-inflammatory cytokines in vaginal fluid might also be a risk factor of preterm birth that was observed to be associated with a decreased vaginal colonization with *Lactobacillus* species (187). In summary, a vaginal bacterial microbiome rich in *Lactobacillus* species is important for the protection against urogenital infections as well as a beneficially balanced cytokine and chemokine profile in the genitourinary tract.

### Genitourinary Tract Archaeome

The archaeal species *Methanobrevibacter smithii* was detected in vaginal samples and was associated with bacterial vaginosis (188, 189). Consequently, the presence of *Methanobrevibacter smithii* was proposed as a biomarker for the diagnosis of bacterial vaginosis (189). Apart from this, there is only little information available on associations between residing archaea and genitourinary tract diseases.

### Genitourinary Tract Mycobiome

Apart from other commensal communities, fungi are a common constituent of the female vaginal microbiome in healthy women (24). The vaginal mycobiome is dominated by the division of Ascomycota, mainly the genus *Candida*, which was present in two-thirds of asymptomatic Estonian women (24, 190). Although candidiasis is one of the most frequent genital diseases, *Candida* species frequently colonize the vagina of healthy women, and only little is known about the influence of vaginal fungal communities on the host’s physiology (191).

### Genitourinary Tract Virome

Most vaginal DNA viruses that are identified today are double-stranded DNA bacteriophages, with eukaryotic viruses constituting only 4% of the total reads (192). Eukaryotic DNA viruses in the vagina are dominated by *papillomavirus* species, but also *herpesviruses*, *polyomaviruses*, and *anelloviruses* can be detected (192–194). Additionally, several groups have identified functional and nonfunctional prophages in the genomes of vaginal bacterial species, suggesting bacteriophages to play a role in shaping the vaginal bacterial microbiome, thereby influencing vaginal health (195–197). Another study also found links between eukaryotic viral and bacterial community composition and the occurrence of bacterial vaginosis (192). In conclusion, there are many potential trans-kingdom interactions between viral and bacterial communities in the vagina and thus indirect and potentially also direct influences of the vaginal virome on health and disease of the genitourinary system.

Endogenous retroviruses are a group of transposable elements that are stably integrated into the genome of their host. They were originally acquired by infection of the host’s germline cells with retroviruses and are vertically transmitted because of their permanent integration (198). The most prominent representatives of human endogenous retroviruses are syncytin-1 and syncytin-2, which are homologous to the surface proteins encoding *env* genes of human endogenous retroviruses (199). They have an essential role in the host’s physiology of the placenta by mediating cell fusion between cytotrophoblasts and placental syncytiotrophoblasts (200). Thus, these relicts of ancient viral infections play a fundamental role in the exchange of nutrients, gases, and hormones between the mother and the fetus. Additionally, they prevent fetal rejection by controlling the maternal immunosuppressive state (201, 202). Hence, these relicts of ancient viral infections were critically involved in mammalian evolution, a prime example of the intimate relationship between microorganisms and their hosts.

Taken together, the bacterial microbiome undoubtfully plays an essential role for the genitourinary tract. However, other microbial kingdoms and their important impact on health and diseases of the genitourinary tract are increasingly evident, appreciated, and also studied.

## Discussion and Outlook

The past decade was characterized by substantial technological progress, allowing researchers to study the mammalian microbiome and its impact on the host in health and disease in a much more precise and mechanistic fashion. As a consequence, it has been established that the microbiota plays a pivotal role in virtually every aspect of mammalian physiology, particularly in the development, maturation, homeostasis, orchestration, and ultimately the function of the immune system (7, 8). By now, it can be considered as textbook knowledge that mammals are metaorganisms and that the combination of the host genome and the overall microbiome including all kingdoms at all epithelial barrier sites largely drives their phenotypes (15). This can be considered the most important conceptual advance in the field of microbiome research during the last decades.

The comparison of conventional SPF mice to germ-free, antibiotic-treated, and gnotobiotic mouse models was pivotal in illuminating the impact of the mammalian microbiome on host physiology. These proof-of-principal studies were essential in illustrating the therapeutic potential lying within microbiome research such as the remarkably diverse set of biologically active compounds produced by the microbiota. As illustrated throughout this review, these compounds can not only influence their corresponding barrier site but also establish axes of communication, thereby exerting crucial systemic effects. Translational microbiome research should aim to identify these compounds and to understand their biological function in health and disease. Among others, this may be a pathbreaking strategy to discover novel microbiota-based drugs (114, 203).

Aside from these important studies, a substantial body of literature emphasizes that the majority of rodent-based data could not be translated into clinical practice (204–211). Recent paradigm-shifting work illustrated that lab mice are too far removed from natural environmental conditions to reliably mirror the physiology of free-living mammals like humans (212–215). This circumstance distorts how the immune system of ultra-clean lab mice develops and functions, leading to false assumptions of how the human immune system works as reviewed elsewhere (216, 217). To address these shortcomings several approaches have been suggested: Cohousing of lab mice with pet store mice (212), sequential infections of lab mice (213), rewilding of lab mice in semi-natural habitats (214), engraftment of wild mouse gut microbiota into lab mice (215) and the transfers of lab mouse embryos into wild mouse surrogate mothers, the so-called “wildling” model (218). Indeed, compared to conventional lab mice, the resulting animals were protected in models of infectious diseases and cancer and displayed an increased translational research value (212, 215). Particularly, wildlings phenocopied the human outcome and could have prevented catastrophically failed clinical trials, where conventional rodent and non-human primate models had failed to predict the human response to harmful drug treatments (218–220). Thus, utilizing these microbially diverse models in translational microbiome research may help to discover novel disease treatment options that cannot be found in conventional mouse models and increase the safety and success rate of bench-to-bedside efforts.

As mentioned above, only a few findings in microbiome research could be directly translated into the clinic so far and many of them originate from human research. For example, there are ideas to use bacterial products in inflammatory skin disease, and promising studies have already proposed a beneficial outcome (159). Moreover, the injection of beta-glucans from fungi cell walls alongside therapeutic antibodies or chemotherapy in cancer treatment shows promising results (221, 222). Another well-known example is allogenic FMT, a potent treatment of antibiotic-refractory *Clostridium difficile* infection (223, 224). Recent studies also showed a beneficial combination of FMT with immunotherapy to overcome the initial resistance to immunotherapy in melanoma patients (225, 226). Allogeneic hematopoietic stem cell transplantation (allo-HSCT) is accompanied by the usage of broad-spectrum antibiotics leading to a low diversity of the gut microbiome which can be successfully treated by autologous FMT (227). This might be a promising treatment option as low-diversity microbiota correlate with increased mortality in allo-HSCT patients (228). Eran Elinav and colleagues published an encouraging exploratory study utilizing vaginal microbiome transplantation to efficiently treat recurrent bacterial vaginosis (229). Even though important steps have already been made, the journey of microbiome research and the successful transfer of microbiota-related therapies into the clinic has only begun.

Thus, besides further technological advances, the key to tap into the full therapeutic potential of translational microbiome research may be: (I) a stronger appreciation of mammals as metaorganisms; (II) the more pronounced investigation of non-bacterial members of the microbiome as well as trans-kingdom interactions at all epithelial barrier sites; (III) a focus on how the bidirectional crosstalk between the host and its microbiota works from a mechanistic standpoint of view, particularly (IV) how microbial biologically active compounds affect the health and disease of the host and (V) take advantage of newly developed translational microbiome research mouse models that more closely resemble the human metaorganism.

This approach will open up a promising window of opportunity to discover novel treatments for a wide range of human diseases of global relevance including transplant rejection, GvHD, cancer, infectious diseases, allergies, autoimmune and inflammatory diseases, psychiatric and neurological disorders as well as cardiovascular diseases.

## Author Contributions

SR wrote the manuscript. SR and SPR designed the figures. SPR supervised and edited the manuscript. All authors contributed to the article and approved the submitted version.

## Funding

SPR was supported by the Deutsche Forschungsgemeinschaft DFG (German Research Foundation): Emmy Noether-Programm RO 6247/1-1, SFB 1160/2 IMPATH and IMM-PACT-Programme for Clinician Scientists, Department of Medicine II, Medical Center – University of Freiburg and Faculty of Medicine, University of Freiburg, 413517907.

## Conflict of Interest

The authors declare that the research was conducted in the absence of any commercial or financial relationships that could be construed as a potential conflict of interest.

## Abbreviations

AD, Alzheimer’s disease; ALD, Alcoholic liver disease; allo-HSCT, Allogeneic hematopoietic stem cell transplantation; ASCA, Anti-*saccharomyces cerevisiae* antibodies; AhR, aryl hydrocarbon receptor; ASD, autism spectrum disorder; CARD, caspase recruitment domain-containing protein; CCR, C-C chemokine receptor; CLEC7A, C-type lectin domain family 7 member A; DC, dendritic cell; DSS, Dextran sulfate sodium; γδT cell, gamma delta T cell; EAE, experimental autoimmune encephalitis; FMT, fecal microbiota transplantation; FXR, farnesoid X receptor; GvHD, Graft-versus-host disease; HCC, hepatocellular carcinoma; HIV, *human immunodeficiency virus*; HSV-2, *herpes simplex virus 2*; IAV, *influenza type A virus*; IL-1RA, IL-1 receptor antagonist; ILC, innate lymphoid cell; IFN, interferon; IL, interleukin; MNP, mononuclear phagocyte; MS, multiple sclerosis; MCMV, murine cytomegalovirus; NAFLD, non-alcoholic fatty liver disease; NKT cell, natural killer T cell; PD, Parkinson’s disease; PD-L1, programmed cell death 1 ligand 1; PRR, pattern recognition receptor; PGE2, prostaglandin E2; RIG-I, retinoic acid-inducible gene I; ROS, reactive oxygen species; T_reg_ cell, regulatory T cell; SFB, segmented filamentous bacteria; SCFA, short-chain fatty acids; SPF, Specific-pathogen-free; STING, Stimulator of interferon genes; T_H_1 cell, T helper 1 cell; T_H_2 cell, T helper 2 cell; T_H_17 cell, T helper 17 cell; TLR, toll-like receptor.
